# Supplementary material for: Interrogating endothelial barrier regulation by temporally resolved kinase network generation
Source: Life Sci Alliance. 2024 Mar 11;7(5):e202302522. doi: 10.26508/lsa.202302522 (PMC10927359; doi:10.26508/lsa.202302522)
Supplement: Supplementary file 6 [file LSA-2023-02522_SdataF4.2_F6.5_FS5.2.pdf]

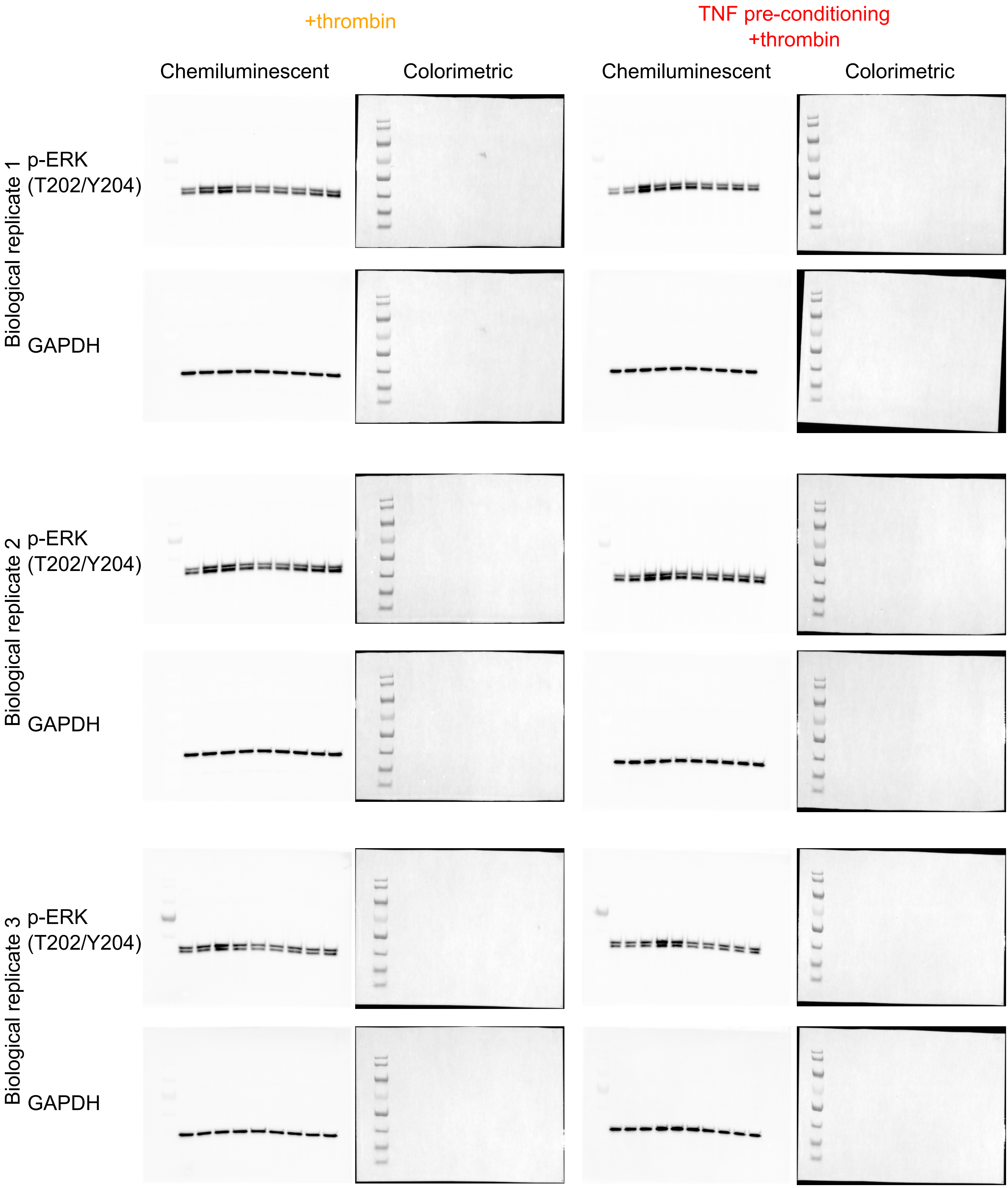
+thrombin
TNF pre-conditioning
+thrombin
Chemiluminescent
Colorimetric
Chemiluminescent
Colorimetric
Biological replicate 1
p-ERK
(T202/Y204)
GAPDH
Biological replicate 2
p-ERK
(T202/Y204)
GAPDH
Biological replicate 3
p-ERK
(T202/Y204)
GAPDH
