# Supplementary figures and images for: Interrogating endothelial barrier regulation by temporally resolved kinase network generation
Source: Life Sci Alliance. 2024 Mar 11;7(5):e202302522. doi: 10.26508/lsa.202302522 (PMC10927359; doi:10.26508/lsa.202302522)

TNF pre-conditioning  
+thrombin

## Colorimetric

p-MK2  
(T334)

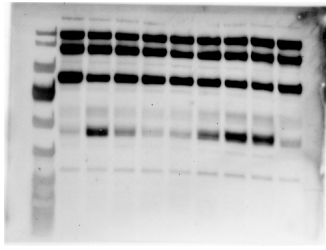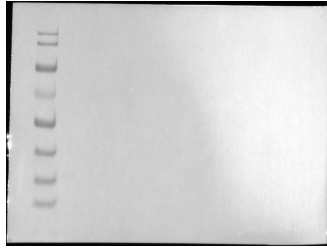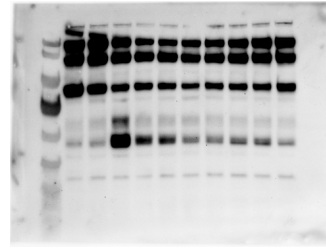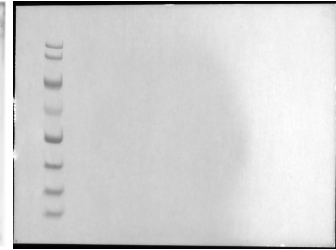

GAPDH

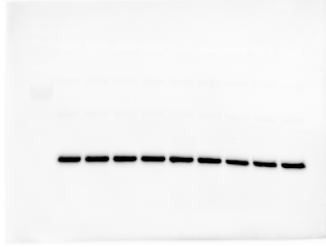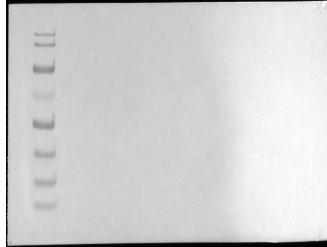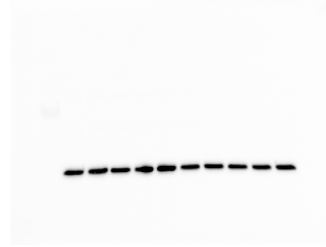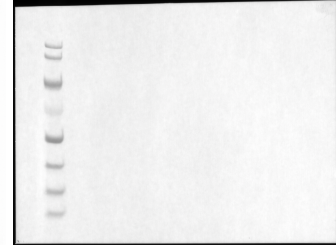

p-MK2  
(T334)

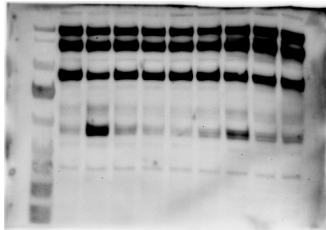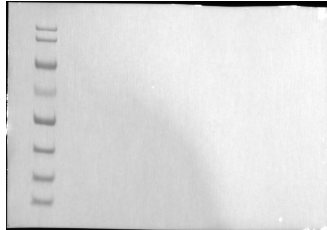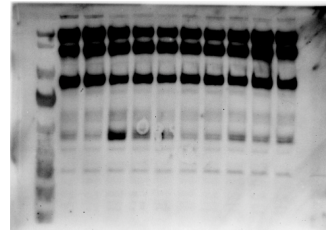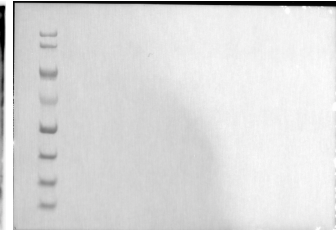

GAPDH

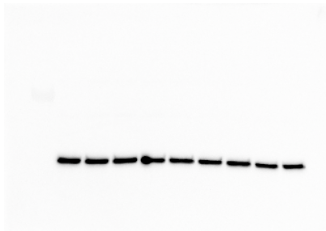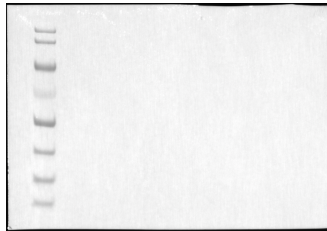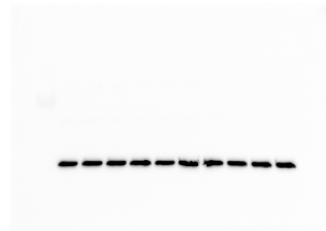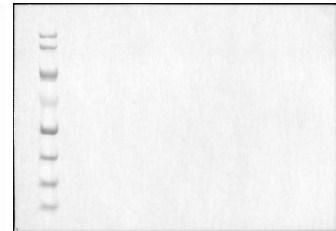

p-MK2  
(T334)

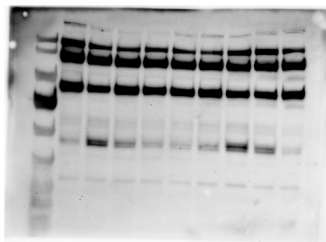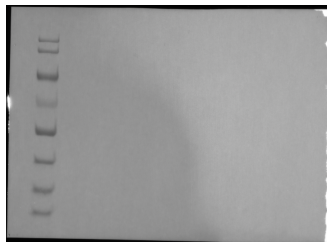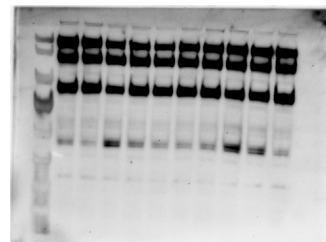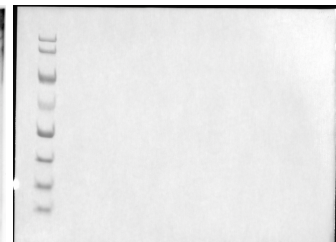

GAPDH

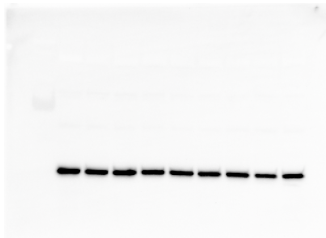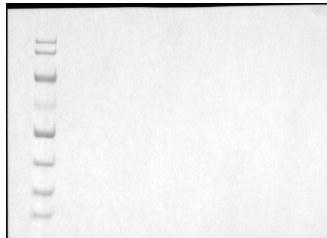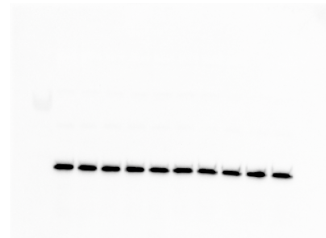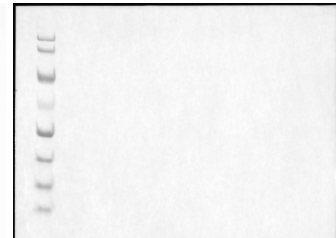

Supplement: Supplementary file 8 [file LSA-2023-02522_SdataF4.4_F5.3_F6.8_FS5.4.pdf]

TNF pre-conditioning  
+thrombin

## Colorimetric

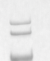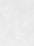[illegible]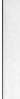

Supplement: Supplementary file 12 [file LSA-2023-02522_SdataF5.3.pdf]

TNF pre-conditioning  
+thrombin

## Colorimetric

p-NIK  
(T559)

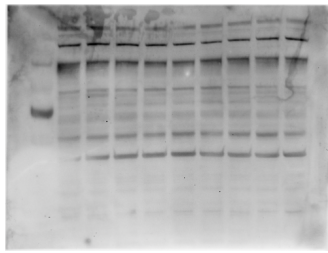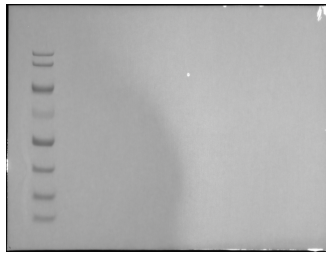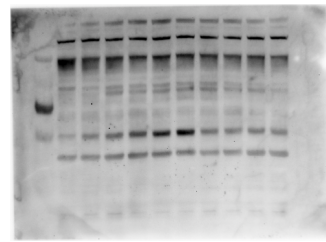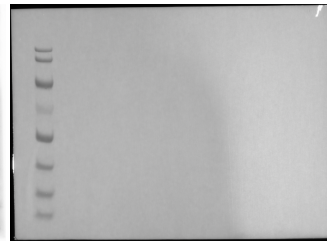

GAPDH

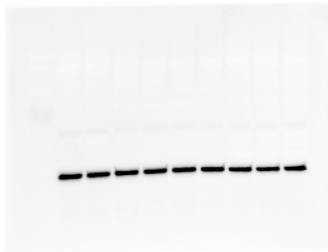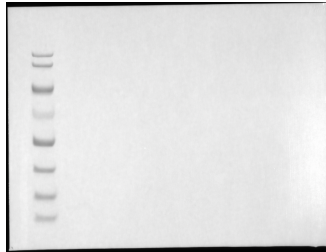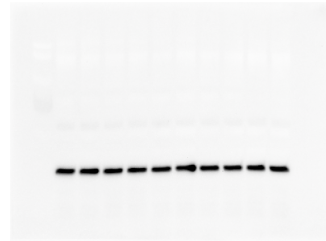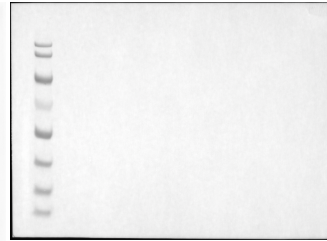

p-NIK  
(T559)

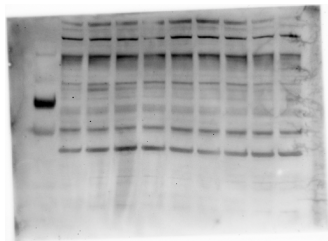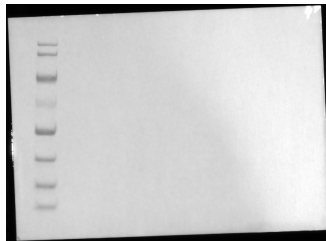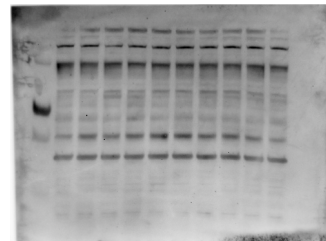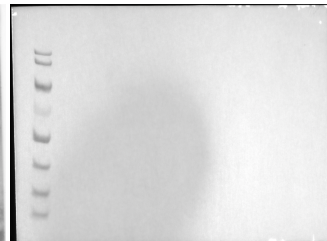

GAPDH

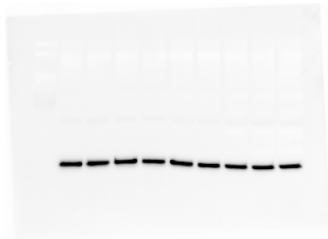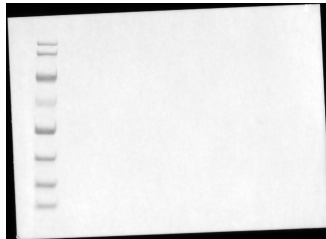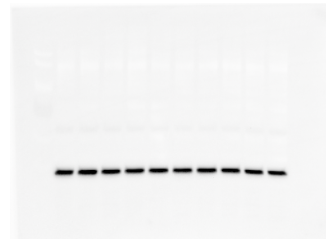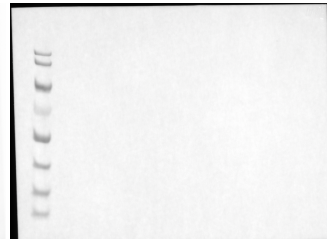

p-NIK  
(T559)

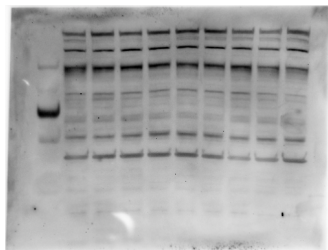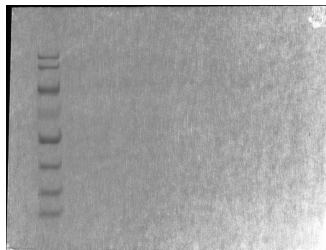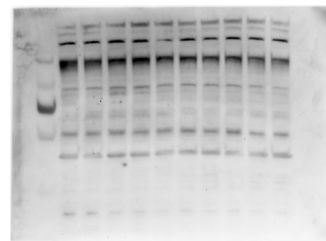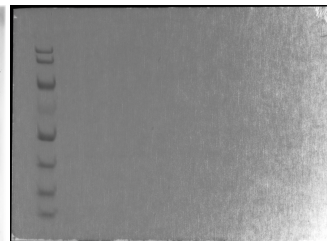

GAPDH

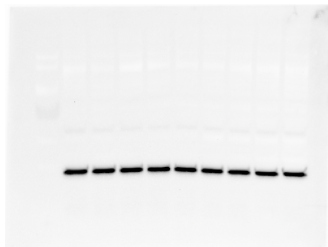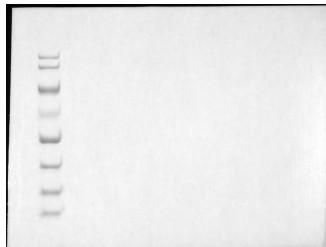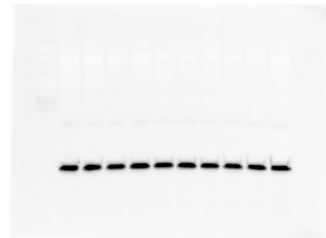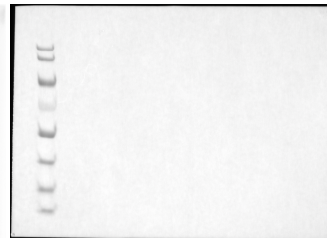

Supplement: Supplementary file 17 [file LSA-2023-02522_SdataF6.6.pdf]
