# Supplementary material for: Interrogating endothelial barrier regulation by temporally resolved kinase network generation
Source: Life Sci Alliance. 2024 Mar 11;7(5):e202302522. doi: 10.26508/lsa.202302522 (PMC10927359; doi:10.26508/lsa.202302522)
Supplement: Supplementary file 10 [file LSA-2023-02522_SdataF4.6_FS5.6.pdf]

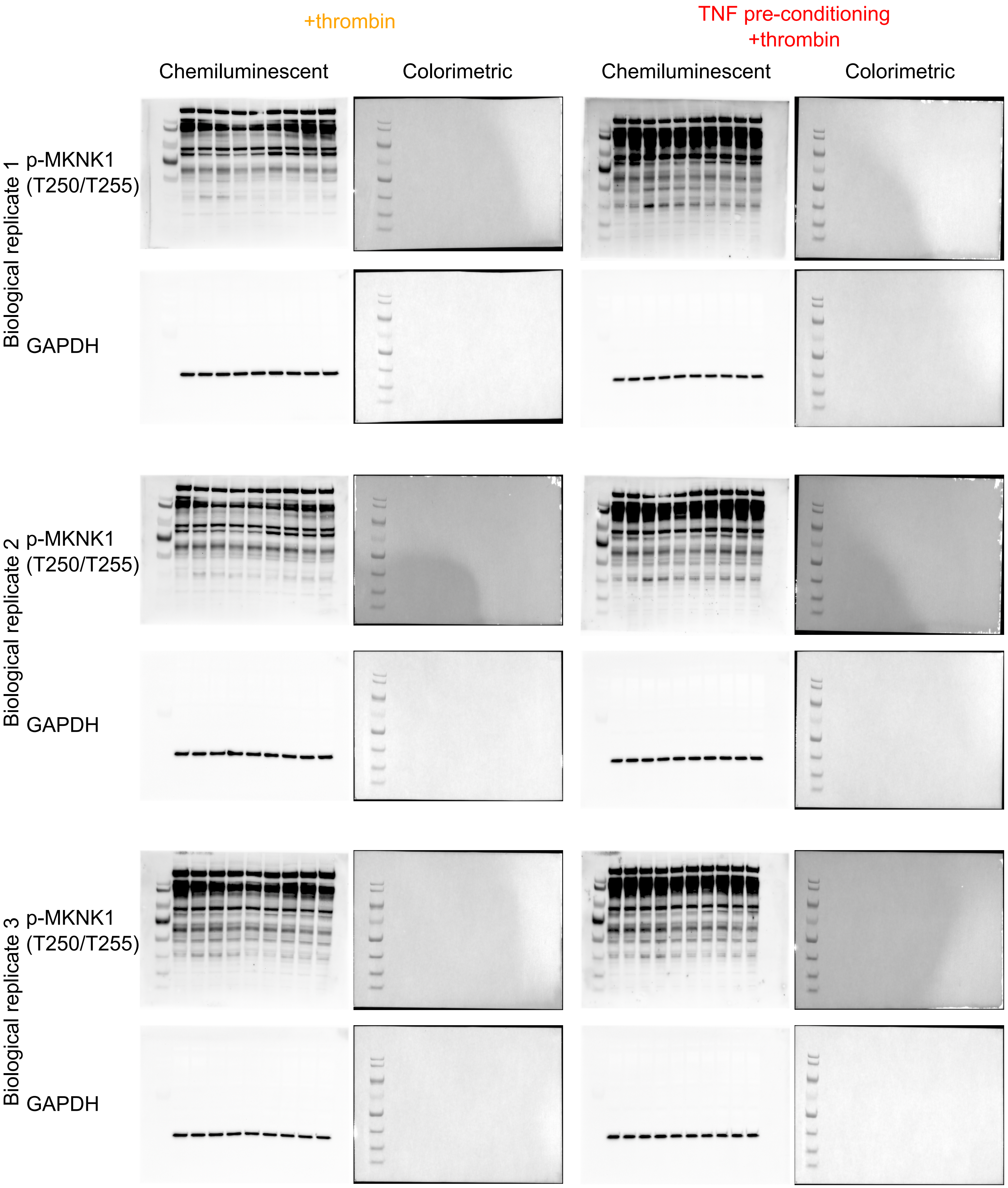
+thrombin
TNF pre-conditioning
+thrombin
Chemiluminescent
Colorimetric
Chemiluminescent
Colorimetric
Biological replicate 1
p-MKNK1
(T250/T255)
GAPDH
Biological replicate 2
p-MKNK1
(T250/T255)
GAPDH
Biological replicate 3
p-MKNK1
(T250/T255)
GAPDH
